# Recent advances in the anti‐aging effects of phytoestrogens on collagen, water content, and oxidative stress

**DOI:** 10.1002/ptr.6538

**Published:** 2019-11-20

**Authors:** Tao Liu, Nan Li, Yi‐qi Yan, Yan Liu, Ke Xiong, Yang Liu, Qing‐mei Xia, Han Zhang, Zhi‐dong Liu

**Affiliations:** ^1^ Institute of Traditional Chinese Medicine Tianjin University of Traditional Chinese Medicine Tianjin China; ^2^ Laboratory of Pharmacology of TCM Formulae Co‐Constructed by the Province‐Ministry Tianjin University of Traditional Chinese Medicine Tianjin China; ^3^ Tianjin State Key Laboratory of Modern Chinese Medicine Tianjin University of Traditional Chinese Medicine Tianjin China; ^4^ Engineering Research Center of Modern Chinese Medicine Discovery and Preparation Technique, Ministry of Education Tianjin University of Traditional Chinese Medicine Tianjin China; ^5^ Information Center Tianjin Polytechnic University Tianjin China; ^6^ Chinese Medical College Tianjin University of Traditional Chinese Medicine Tianjin China

**Keywords:** collagen, estrogen, oxidative stress, phytoestrogens, skin aging, water content

## Abstract

Skin undergoes degenerative changes as it ages, which include the loss of elasticity, reductions in the epidermal thickness and collagen content, elastic fiber degeneration, and increased wrinkling and dryness. Skin aging can be significantly delayed by the administration of estrogen. Estrogen deficiency following menopause results in atrophic skin changes and the acceleration of skin aging. Estrogen administration has positive effects on human skin by delaying or preventing skin aging manifestations, but the use of estrogen replacement is a risk factor for breast and uterine cancer. Phytoestrogens are a large family of plant‐derived molecules possessing various degrees of estrogen‐like activity; they exhibit agonist or antagonist estrogenic properties depending on the tissue. These molecules could be ideal candidates to combat skin aging and other detrimental effects of hypoestrogenism. In this paper, we review the effects of phytoestrogens on human skin and the mechanisms by which phytoestrogens can alleviate the changes due to aging.

## 1 INTRODUCTION

The skin is the largest organ in the body. Like all other tissues, it undergoes degenerative processes with aging. Skin helps in body protection and temperature regulation and acts as an organ of sensation (Nagula & Wairkar, 2019). Skin aging includes a loss of elasticity, a reduction in epidermal thickness and collagen content, elastic fiber degeneration, and increased wrinkling and dryness. The protective functions of the skin become compromised, and aging is associated with impaired wound healing, hair loss, pigmentary changes, and skin cancer (Thornton, 2013).

Skin is a target organ of hormones. Estrogens have a profound influence on skin. Estrogens can significantly modulate skin physiology, targeting keratinocytes, fibroblasts, melanocytes, hair follicles and sebaceous glands, and improve angiogenesis, wound healing, and immune responses (Thornton, 2013). The skin fibroblasts have been reported to biosynthesize/secrete over 998 proteins (Waldera et al., 2015). Estrogen's effects are particularly pronounced in the skin where cutaneous changes post‐menopause are well documented (Emmerson & Hardman, 2012). Ovarian estrogen represents a majority of the estrogens in women, which decline with aging and especially menopause (Lephart, 2018). Estrogen deficiency following menopause results in atrophic skin changes and the acceleration of skin aging. Skin thickness is reduced by 1.13% and collagen content is reduced by 2% per postmenopausal year in menopausal women (Brincat et al., 1987). Types I and III skin collagens are thought to decrease by as much as 30% in the first 5 years after menopause (Affinito et al., 1999; Brincat et al., 1985). These effects in elderly females correlate with the period of estrogen deficiency rather than chronological age (Affinito et al., 1999; Brincat et al., 1985; Brincat et al., 1987). Estrogen insufficiency also decreases the defense against oxidative stress. A decrease in collagen causes the skin to become thinner, and decreases elasticity, increases wrinkling, increases dryness, and reduces vascularity (Thornton, 2013). These skin changes can be reversed by estrogen replacement, which increases keratinocyte proliferation, epidermal thickness, epidermal hydration, and skin elasticity, reduces skin wrinkles, augments the content and quality of collagen, and increases the level of vascularization (Stevenson & Thornton, 2007; Thornton, 2002, 2005). Estrogen administration has positive effects on human skin, but the use of estrogen replacement is a risk factor for breast and uterine cancer. Phytoestrogens are a large family of plant‐derived molecules possessing various degrees of estrogen‐like activity. They can bind both estrogen receptors alpha and estrogen receptors beta and act as both estrogen agonists and antagonists (Hwang et al., 2006). Phytoestrogens are considered to be naturally‐occurring selective estrogen receptor modulators (SERMs) and are potential contenders to provide a natural estrogen replacement in postmenopausal women.

**Figure 1:**
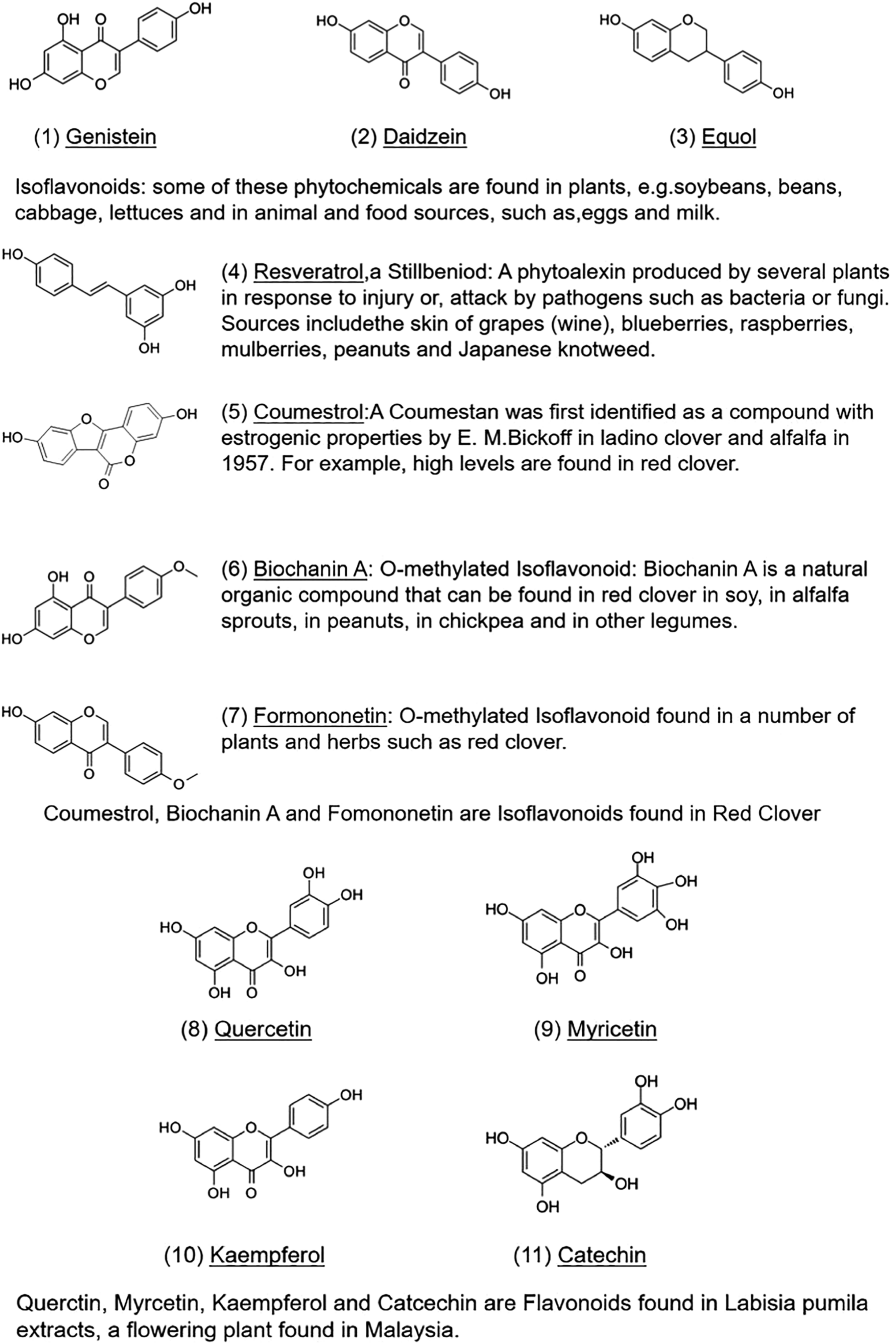
Molecular structure of phytoestrogens

Phytoestrogens have similar chemical structures to the mammalian estrogen, estradiol (Rietjens, Sotoca, Vervoort, & Louisse, 2013). Phytoestrogens can bind to ERα and ERβ, but most compounds possess a preference for ERβ (Paterni, Granchi, Katzenellenbogen, & Minutolo, 2014; Younes & Honma, 2011). ERα and ERβ move from the cytoplasm to the nucleus when binding to ligand‐activated nuclear transcription factors that enhance target gene transcription (Sirotkin & Harrath, 2014). Furthermore, phytoestrogens also bind to membrane estrogen receptors that are coupled to cytosolic signal transduction proteins (Figure 1). These membrane estrogen receptors initiate signal cascades directly via conventional second messengers, which include adenylate cyclase, cyclic adenosine monophosphate (cAMP), phospholipase C, protein kinase C, and mitogen‐activated protein kinase (MAPK), producing rapid responses to phytoestrogens (Sirotkin, 2016; Yanagihara et al., 2014).

Phytoestrogens exert an anti‐aging effect on the skin via estrogen receptors (Gopaul, Knaggs, & Lephart, 2012). They increase the collagen content (Chua, Lee, Abdullah, & Sarmidi, 2012), the production of hyaluronic acid (Patriarca et al., 2013), and extracellular matrix proteins (Gopaul et al., 2012). Phytoestrogens show protection against oxidative stress. Skin aging can be significantly delayed by the administration of estrogen, selective estrogen receptor modulators, and phytoestrogens (Thornton, 2013).

## 2 EFFECTS ON COLLAGEN

The accumulation of collagen breakdown products is a hallmark of aging skin and results in a decrease in mechanical tension that leads to wrinkles. Age‐related differences in the amount and structure of proteoglycans that bind to collagen also determine the mechanical properties of the skin (Jackson, Greiwe, & Schwen, 2011). There are two types of collagen change during the aging process. Type I collagen represents the predominant collagen type in adult human skin, whereas type III collagen, also widely distributed throughout the body, predominates in fetal tissues. For the first time, an increase in mainly type III collagen indicates the distinct modulatory effects of estrogen on collagen tissue (Schmidt, Binder, Demschik, Bieglmayer, & Reiner, 1996). In estrogen‐deficient women, skin thickness and collagen content is reduced. Types I and III skin collagens are thought to decrease, which parallels the reduction in bone mass observed in postmenopausal women. This decrease in skin thickness and collagen content in elderly women correlates with the period of estrogen deficiency rather than chronological age. A difference in collagen subtypes has also been recognized in postmenopausal women; compared with premenopausal women, postmenopausal women demonstrate a decrease in collagen types I and III and a reduction in the type III/type I ratio within the dermis, again correlating with the period of estrogen deficiency rather than chronological age (Thornton, 2013).

It has been reported that estrogen is effective in the treatment of aging skin, and an improvement in skin elasticity and wrinkle depth is observed after 6 months of treatment in premenopausal women with skin aging symptoms (Calleja‐Agius, Brincat, & Borg, 2013). Current studies have shown that estrogen treatment prevents the loss of the collagen I peptide and increases the expression of type III collagen (Voloshenyuk & Gardner, 2010). The same study also demonstrated that estrogen increased tropoelastin and fibrillin, which may be associated with an increase in elastic fibers. Transforming growth factor β (TGF‐β) is a growth factor that stimulates fibroblast proliferation and extracellular matrix (ECM) secretion, which can affect angiogenesis and epithelialization of the skin. TGF‐β treats skin aging by enhancing the production of subcutaneous VEGF and thereby increasing the thickness of collagen. Estrogen can enhance the expression of TGF‐β to delay skin aging through the above mechanisms (Thornton, 2013). Matrix metalloproteinases (MMPs) induce skin aging by degrading collagen. There is increasing evidence that the expression of MMPs is controlled by tissue inhibitor of metalloproteinase (TIMP). TIMP is a tissue inhibitor of MMPs that inhibits MMPs and thereby inhibits collagen degradation. In addition to its inhibitory effects on most known MMPs, the encoded TIMP proteins are capable of promoting cell proliferation in a variety of cell types and may also have anti‐apoptotic functions. Estrogen can upregulate the expression of TIMPs to downregulate the expression of MMPs, which reduces the degradation of collagen to protect the skin (Kassira et al., 2009; Nemitz et al., 2015).

The estrogen‐like effects of some plants were first described by Loewe et al. in 1927. Phytoestrogens are classified into three categories: isoflavones, coumestans, and lignans. The most important and abundant isoflavones in soybeans are genistein and daidzein, which display some similarity to the mammal estrogen molecule and are found, inter alia, soybeans, lentils, and red clover. The most important isoflavones are genistein and daidecin. Coumestans only occur in the sprouts of legumes. Their structural similarity to 17β‐estradiol explains the estrogen‐like effects, which may be traced back to the interaction of these substances with the estrogen receptor (Sator, Schmidt, Rabe, & Zouboulis, 2004). A controlled, open European multicenter study examined the effect of a cosmetic cream preparation that includes isoflavone (Novadiol). Compared to untreated skin areas, isoflavones significantly reduced facial wrinkles by 22% and skin loss by 24% by affecting collagen synthesis and reducing enzymatic collagen degradation. These data show that isoflavones can significantly reduce wrinkles and significantly improve the clinical manifestations of the skin (Bayerl & Keil, 2002; Sator, 2006).

Genistein (an aglycone), which is an isoflavone found in low concentrations in soybeans and in increased amounts in certain fermented soy foods, has been recently considered an ideal natural selective estrogen receptor modulator (SERM). Genistein has been tested in anti‐aging cosmetic preparations and has shown interesting results for skin elasticity, photoaging, and skin cancer prevention. Cosmetic creams containing genistein (an aglycone) have been used to improve skin dryness and wrinkles (Rona, Vailati, & Berardesca, 2004). Researchers have explored the mechanism by which genistein delays skin aging, and it was found that genistein increased the thickness of skin collagen by inducing the expression of subcutaneous VEGF and increasing TGF‐β in the skin. Genistein also inhibited MMPs by increasing TIMP protein levels, in turn decreasing the degradation of collagen. Therefore, genistein can significantly increase the thickness of collagen and delay skin aging (Polito et al., 2012).

Daidzein is an isoflavone with extensive nutritious value and is mainly extracted from soy plants. It is also called phytoestrogen due to its structural similarity to the human hormone estrogen. Daidzein is reported to play a significant role in the prevention and treatment of a variety of diseases such as cancer, cardiovascular disease, diabetes, osteoporosis, skin disease, and neurodegenerative disease. This pharmacological activity is attributed to various metabolites including equol and trihydroxy isoflavone (Meng‐Yao et al., 2016). Kim, Hong, and Lee (2014) investigated whether daidzein, a potent isoflavone, displays phytoestrogen activity and induces transcriptional changes in extracellular matrix components in dermal fibroblasts. The estrogenic receptor‐dependent transcriptional activity increased in a dose‐dependent manner when treated with daidzein, with a maximum of 2.5‐fold induction at 10 μg/mL of daidzein compared with the nontreated control. In addition, daidzein significantly increased the expression of collagen type I, collagen type IV, elastin, and fibrillin‐1 in human dermal fibroblasts (Lee, 2014).

Coumestrol is a polyphenol with promising therapeutic applications as phytoestrogen and antioxidant. The presence of two hydroxyl groups on its chemical structure, with orientation analogous to estradiol, is responsible of both, its antioxidant capacity and its estrogenic activity (Bianchi et al., 2018; Montero et al., 2019; Simons, Gruppen, Bovee, Verbruggen, & Vincken, 2012). Under stresses such as germination, fungal infection, or chemical elicitors, coumestrol is produced as a phytoalexin through the conversion of daidzein, the aglycon of daidzein (Simons et al., 2011). Researchers have found the potential protective effect of coumestrol against ultraviolet B (UVB)‐induced skin photoaging and aimed to uncover the direct molecular target of coumestrol. A whole human kinase profiling assay identified FMS‐like tyrosine kinase 3 (FLT3) as a novel target protein of coumestrol in the UVB‐induced signaling pathway in the skin. Coumestrol suppresses FLT3 kinase activity and, subsequently, the Ras/mitogen‐activated protein kinase/extracellular signal‐regulated kinase (Ras/MEK/ERK) and Akt/p70 ribosomal S6 kinase pathways. Inhibition of these pathways leads to inhibition of activator protein 1 (AP‐1) activity, which in turn reduces the transcription of the MMP‐1 gene to reduce its degradation of collagen (Park et al., 2015). Coumestrol has certain side effects on the reproductive system due to its estrogen receptor binding characteristics (Solak et al., 2014). Some researchers studied the effects of coumarin phytoestrogens on the reproductive system of mice, and found that reproductive abnormalities occurred in mice (Burroughs, Mills, & Bern, 1990; Lee, Yuk, Park, & Bae, 2013; Moon et al., 2009; Solak et al., 2014). Clinically, it has also been reported that phytoestrogens have some negative effects on reproduction in humans (Milligan et al., 1999; North & Golding, 2000).

Equol is found in white cabbage and is produced by intestinal flora in humans and animals via the conversion from daidzein (Hounsome, Hounsome, Tomos, & Edwards‐Jones, 2009; Setchell et al., 2005). Equol has recently caught the interest of researchers because of its powerful antioxidant activity and its unique molecular and biochemical messenger properties with implications in treating age‐related diseases (Rufer & Kulling, 2006; Setchell et al., 2005). Of particular interest, equol has an affinity for ERβ, which is abundant in the keratinocytes of the epidermis and the fibroblasts of the dermis (Thornton et al., 2003). Researchers have used human dermal models to study the effects of equol on the skin. The results show that equol may be used locally to treat and prevent skin aging by enhancing the ECM component in the skin, specifically by stimulating the production of type I collagen, type III collagen and the protein elastin (ELN) while downregulating MMPs. In addition, equol can also increase the expression of skin antioxidants and prevent androgenic damage to the skin (Gopaul et al., 2012). In skin, equol has been shown to improve dermal health by direct and downstream influences at several different steps of the oxidative stress cascade, while at the same time inhibit MMP's actions and simultaneously stimulate collagen and elastin. Lephart found equol's action of stimulation of: Nrf2, antioxidant/detoxifying enzymes, and extra cellular matrix proteins along with DNA and tissue repair and, equol's inhibition of: nuclear factor‐κB (NF‐κB), pro‐inflammatory biomarkers, and MMPs (Lephart, 2016).

Resveratrol is a phytoestrogen component that has attracted increasing research attention to understand its properties and benefits for various diseases and conditions, including skin health. Resveratrol has a similar chemical structure to 17β‐estradiol and has estrogen receptor (ER) binding characteristics that favor affinity to the ERβ subtype. The human skin has a large amount of ERβ through which resveratrol can act. Researchers have found that resveratrol can increase the production of collagen and elastin and inhibit MMPs by stimulating TIMP1, thereby inhibiting the decomposition of collagen and exerting its anti‐aging effect (Giardina et al., 2010; Lephart, 2017). Lephart et al. found that resveratrol can ameliorate the aging of human skin by significantly stimulating sirtuin (silent mating type information regulation 2 homolog) 1 (SIRT1), extracellular matrix proteins, such as collagens and elastin, and antioxidants while significantly inhibiting inflammatory and dermal‐aging biomarkers. Resveratrol's anti‐aging protection altered specific dermal biomarkers through a variety of mechanisms to protect the skin (Lephart, Sommerfeldt, & Andrus, 2014).

Plant extracts, such as red clover, which contain high levels of isoflavones, have been used to reduce menopausal symptoms and have been shown to reduce bone loss in healthy women. Clara Circosta et al. investigated the effects of red clover isoflavones on skin aging. The histology of the skin, skin thickness, and the amount of total collagen determined by a colorimetric method were studied in ovariectomized (OVX) rats after treatment for 14 weeks. In OVX rats, the thickness and keratinization of the epidermis were reduced; glands were fewer in number and the vascularity was poor; and the distribution and morphology of the collagen bundles and elastic fibers were altered. Whereas the skin of the OVX rats treated with red clover isoflavones appeared well organized with a normal epidermis with uniform thickness and regular keratinization, the vascularity, collagen, and elastic fibers were well developed. The amount of collagen significantly increased in the treated group in comparison with the control group. These findings suggest that red clover isoflavones are an effective treatment of anti‐skin aging induced by estrogen deprivation (Circosta, De Pasquale, Palumbo, Samperi, & Occhiuto, 2006).

*Labisia pumila* is a traditional herb widely used for centuries as a postpartum medication. It is believed that this plant contains phytoestrogens. These are likely estrogen receptor modulators that displace 17β‐estradiol binding to antibodies raised against estradiol, making these phytoestrogens similar to other estrogens, such as esterone and estradiol (estrogen‐like compounds) (Chua et al., 2011). A study found that *L. pumila* can reduce MMP production, and promote skin collagen synthesis and the growth of human skin fibroblasts and keratinocytes to improve skin aging caused by estrogen deficiency. The herbal extract also has the ability to protect human skin from reactive oxygen species (ROS) attacks generated by UVB exposure. This is mainly due to the presence of bioflavonoids and phenolic acids in the plant extract. These compounds possess a rejuvenating effect on cell proliferation and morphology as well as oxidation‐related diseases, including aging. In addition, the *L. pumila* extract was likely to reduce the secretion of proinflammatory cytokines, which are usually associated with various skin diseases and the progression of photodamaged skin (Chua et al., 2012).

## 3 EFFECT ON WATER CONTENT

Healthy skin needs a lot of water. The water content of the skin depends on the evaporation rate through the skin and the hydration of the epidermis (Brincat, Baron, & Galea, 2005). Glycosaminoglycans in the dermis, especially heparan sulfate and versican, are essential for hydration of the skin because they contain large amounts of water. As the amount of glycosaminoglycans decreases with age, the hygroscopicity of the skin also decreases (Calleja‐Agius et al., 2013). Hyaluronic acid (HA) is abundant in the dermis and is believed to contribute to moisture content, skin atrophy, and the spread of soluble factors and nutrients (Stern & Maibach, 2008). In addition, HA can support the proliferative phenotype of fibroblasts and may oppose apoptosis (Dai et al., 2007; Yoneda, Yamagata, Suzuki, & Kimata, 1988). HA is synthesized at the plasma membrane by hyaluronan synthases (HASs) (Itano & Kimata, 2002). These enzymes extrude HA into the extracellular space after the assembly of uridine diphosphate (UDP)‐glucuronic acid and uridine diphosphate‐*N*‐acetyl‐d‐glucosamine into a growing chain of (1–3)‐linked d‐glucuronic acid and *N*‐acetyl‐d‐glucosamine disaccharides (Pontius & Smith, 2011; Rock et al., 2012). Repeated disaccharides are linked by hexosamine (1–4) bonds to form high molecular weight HA that reaches up to 107 Da and 20 μm (Toole, 2004). HAS is expressed in relatively low copy numbers per cell but can rapidly produce large amounts of HA (Bourguignon, Gilad, & Peyrollier, 2007; Karousou et al., 2010; Vigetti et al., 2009).

Research shows that the positive effects of estrogens on the water content of the skin may be due to dermal and epidermal components. Estrogen is necessary to maintain glycosaminoglycans and hyaluronic acid in the skin. Estrogen stimulation increases the levels of glycosaminoglycans and hyaluronic acid, which contribute to an increased water content in the dermis. The water content of the epidermis is related to the thickness of the epidermis and the amount of natural moisturizing factors. Another study showed that estrogen induced the expression of the epidermal growth factor in keratinocytes, which stimulated the expression of HAS in dermal fibroblasts. The synthesis of HA in the dermis increased, which enhanced the hygroscopicity of the skin (Calleja‐Agius et al., 2013; Pontius & Smith, 2011; Rock et al., 2012; Schmidt et al., 1996).

Phytoestrogens have structures that are very similar to endogen estradiol derived from plants (Desmawati & Sulastri, 2019). Some phytoestrogens can resist skin aging by improving skin moisture levels. Soybean ingredient has an estrogen‐like effect. A study found that the most important and abundant isoflavones in soybeans stimulated the production of collagen and HA by human dermal fibroblasts in vitro. Soy isoflavone significantly ameliorated skin aging by increasing HA and HAS. These findings indicate the potential of soy isoflavone to prevent skin aging (Bhattacharyya, Higgins, Sebastian, & Thomas, 2009; Huang et al., 2010; Miyazaki et al., 2004).

Transepidermal water loss (TEWL) is a measure of the water permeability of the skin barrier that is determined by measuring the water vapor pressure gradient on the surface of the skin to indicate the loss of water. Measurements of TEWL may be useful to identify skin damage caused by certain chemicals and can also be used to assess the degree of skin aging (Szczepanik et al., 2018). Soy isoflavone extract ISO‐1 (containing 12 soy isoflavones) from soybean cake was demonstrated to prevent skin damage caused by UVB exposure. The researchers found that soy isoflavones (daidzin, genistin and glycitin) could inhibit UVB‐induced death of human keratinocytes and reduce the level of desquamation, TEWL, erythema, and epidermal thickness in mouse skin (Huang et al., 2010; Patriarca et al., 2013).

Recently, more interest has been expressed in the effects of soymilk products and their anti‐aging effects on the skin (Sudel et al., 2005; Varani, Kelley, Perone, & Lateef, 2004). Bhattacharyya TK et al. found that the topical use of soy cream on the skin of mice can significantly increase the amount of HA compared with the control group. The phytoestrogen‐rich soybean component can delay skin aging by improving skin moisture content (Bhattacharyya et al., 2009).

## 4 EFFECT ON OXIDATIVE STRESS

ROS include the superoxide anion (O^2−^), hydrogen peroxide (H_2_O_2_), and hydroxy radicals (˙OH). They cause severe damage to the ECM and the structure of DNA, proteins, and lipids (Ismail, Pravda, Li, Shih, & Dallabrida, 2010). Skin aging is thought to be driven by an increased in‐situ production of ROS, which result from both a disturbance of mitochondrial function and acute stress responses to different environmental insults including solar radiation. ROS turn on cellular and molecular mechanisms that accelerate skin aging including upregulation of transcription factors such as AP‐1 and NF‐κB. AP‐1 is one of the prominent transcription factors responsible for the production of MMPs, the enzymes that break down collagen. The essential role of MMPs in promoting premature skin aging has been demonstrated in pivotal scientific studies. Furthermore, multiple studies have shown that the decrease in collagen production is associated with AP‐1 and may involve the cytokine TGF‐β. This cumulative loss of dermal collagen is believed to be the primary cause of wrinkling. Likewise, NF‐κB is paramount in the production of pro‐inflammatory mediators that contribute to skin aging (Farris, Krutmann, Li, McDaniel, & Krol, 2013; Lephart, 2016). The accumulation of these changes constitutes the basis of cell aging. Estrogen is a potent direct antioxidant and indirect inducer of antioxidant enzymes. Estrogen deficiency is strongly linked with an altered oxidative state. Estrogen has a key role in skin aging. Estrogen deficiency in the perimenopausal years can accelerate skin aging. Oxidative stress decreases procollagen I synthesis in human fibroblasts, while estrogen significantly increases the synthesis of procollagen I. Estrogen can increase the viability of fibroblasts and keratinocytes, which are affected by H_2_O_2_. Furthermore, estrogen is able to counteract H_2_O_2_‐mediated lipoperoxidation and DNA oxidative damage in skin cells. The physiological concentration of estrogens is able to increase cell viability, which is reduced by ROS, and protect human skin cells by decreasing oxidative damage. The presence of estrogen may also protect skin cells against oxidative damage, and the dramatic lowering of estrogen levels during menopause could render skin more susceptible to oxidative damage (Bottai, Mancina, Muratori, Di Gennaro, & Lotti, 2013). In OVX rats, oxidized glutathione, lipid peroxidation, and mitochondrial DNA damage are increased significantly. Estrogen or phytoestrogen can reverse these changes (Baeza, Fdez‐Tresguerres, Ariznavarreta, & De la Fuente, 2010). Additionally, keratinocytes isolated from aged female rats exhibit high lipoperoxides and increased caspase 3 and caspase 8, the levels of which can also be reversed by estrogen or phytoestrogen (Tresguerres et al., 2008).

S‐equol, a gut bacterial metabolite of soy daidzein, is a potent antioxidant that selectively binds to ERβ. Equol and 17β‐estradiol have similar chemical structures/confirmations and molecular weights (Lephart, 2016). Equol is a superior antioxidant (Arora, Nair, & Strasburg, 1998; Mitchell et al., 1998). Equol has greater antioxidant activity (i.e., oxidative damage to lipid membranes, etc.) compared to genistein (Mitchell et al., 1998; Rufer & Kulling, 2006). S‐equol reduces oxidative stress in skin by enhancing the expression of antioxidant enzymes. S‐equol binds to ERβ and activates the estrogen response element (ERE), which in turn promotes the dissociation of Nrf2 and Keap1. Nrf2 enters the nucleus and activates the antioxidant response element (ARE), which enhances the transcription of antioxidant enzymes, such as superoxide dismutase (SOD) and glutathione peroxidase (GSH‐Px) (Kim et al., 2014). As discussed by Jackson et al., equol may increase Nrf2 levels and/or bind to the estrogen‐responsive elements (EREs) in the promoter region the Nrf2 gene and/or increase gene expression of other antioxidant genes (Jackson et al., 2011). In support of this concept, Zhang et al. showed that S‐equol provided protection against peroxide‐induced endothelial cell apoptosis by activation of estrogen receptor and Nrf2/ARE signaling pathways (Zhang et al., 2013). Finally, Froyen and Steinberg reported that racemic equol increased the expression of the xenobiotic metabolizing enzyme quinone reductase (both mRNA and protein levels) via similar molecular mechanisms involving ERβ and Nrf2 (Froyen & Steinberg, 2011).

Genistein, the main isoflavone contained in soybeans and fermented soy foods, is a promising anti‐aging and anticarcinogenic agent for skin due to its antioxidant properties. It has been suggested for topical use to prevent skin aging after menopause (Jurzak & Adamczyk, 2013; Polito et al., 2012; Wang et al., 2019). Paola Savoia et al. found that genistein was able to improve mitochondrial membrane potential and increase NO release under physiological conditions. It can also hinder the decrease in mitochondrial membrane potential and the increased NO release caused by H_2_O_2_. Genistein reduces ROS release by increasing GSH levels, which is accompanied by an increase in the cell viability and proliferation rates. It is indicated that genistein can achieve anti‐aging effects by protecting the skin from oxidative stress (Savoia et al., 2018).

Resveratrol, an antioxidant polyphenol from red wine, has been the subject of intense interest in recent years due to a range of unique anti‐aging properties. Resveratrol has been reported to be a strong inhibitor of peroxidation (Baxter, 2008). Resveratrol can activate ERα and ERβ, which are potent antioxidants with strong anti‐inflammatory properties (Rona et al., 2004). Botanical antioxidant compounds as a key ingredient in skin care products have received recent attention and validation of efficacy. Resveratrol has been demonstrated to act on cellular signaling mechanisms related to UV‐mediated photoaging, including MAP kinases, NF‐κB, and matrix metalloproteinases (Table 1). Resveratrol lowers levels of reactive oxygen species in UVA‐exposed HaCaT keratinocytes in a dose‐dependent manner, and electron microscopy confirmed that ultrastructural changes could be prevented (Baxter, 2008). SIRT1 is a member of a highly conserved gene family (sirtuins) encoding NAD^+^‐dependent deacetylases (Vaziri et al., 2001; Luo et al., 2001). SIRT1 plays protective role in UV‐induced skin cell damage. Both UV and H_2_O_2_, two major factors of skin cell damage, down‐regulate SIRT1 in a time‐ and dose‐dependent manner. UV radiation and H_2_O_2_ induce p53 acetylation in cultured skin keratinocytes and MEFs cells, SIRT1, as a deacetylase, negatively regulate UV‐induced p53 acetylation. SIRT activator, resveratrol which has been considered an important antioxidant, protects against UV‐ and H_2_O_2_‐induced apoptotic cell death (Cao et al., 2009). In vitro studies by Farris et al. have demonstrated that resveratrol effectively down regulates ROS‐induced increases in AP‐1 and NF‐κB and thus serves a key role in preserving dermal collagen and reducing skin inflammation (Farris et al., 2013). A recent study demonstrated that resveratrol upregulates mitochondrial SOD in cultured human lung fibroblasts and human neuroblastoma cells (Robb & Stuart, 2011). Resveratrol mainly upregulates mitochondrial SOD via ERβ. Furthermore, the effect of resveratrol could be abolished by the ER antagonist ICI 182780. This indicates that resveratrol can exert its antioxidative stress through ERβ2 (B. Yingngam; Jitsanong, Khanobdee, Piyachaturawat, & Wongprasert, 2011; Yu et al., 2009).

**Table 1:** Mechanism of phytoestrogens use in anti‐aging

Source of phytoestrogens	Composition of phytoestrogens	Effect/mechanism		
		Effects on collagen	Effects on water content	Effects on oxidative stress
Soy	Genistein aglycone	① Increase collagen by inducing VEGF and improve the TGF‐β in the skin. ② Reduces collagen degradation by inhibiting MMPs and increasing TIMP. [OVX rats (Polito et al., 2012; Rona et al., 2004)]	Increase the amount of HA to improve the skin moisture content. [OVX rats (Polito et al., 2012; Rona et al., 2004)] Reduce the level of TEWL. [ICR‐Foxn/^*nu*^ mice (Huang et al., 2010)]	Improve mitochondrial membrane potential and reduce ROS release by increasing GSH level to delay skin aging. [Human fibroblasts and keratinocytes (Savoia et al., 2018)]
	Daidzein	Increase the expressions of collagen type I, collagen type IV, elastin, and fibrillin‐1. [Human dermal fibroblasts (Lee, 2014)]	Reduce the level of TEWL. [ICR‐Foxn/^*nu*^ mice (Huang et al., 2010)]	–
	Coumestrol	Suppresses FLT3, Ras/MEK/ERK and Akt/p70 ribosomal S6 kinase pathway to inhibit AP‐1, reduce the MMP‐1 and reduce collagen degradation of. [Human dermal fibroblasts (Park et al., 2015)]	–	–
	Soy cream	–	Increase the amount of HA to improve the skin moisture content. [Hairless mice (Bhattacharyya et al., 2009)]	–
	Equol	Stimulate the production of type I collagen, type III collagen and ELN protein, down‐regulating MMPs [Human monolayer dermal fibroblast (Gopaul et al., 2012)] Stimulation of Nrf2, antioxidant/detoxifying enzymes, and extra cellular matrix proteins along with DNA and tissue repair, inhibition of: NF‐κB, pro‐inflammatory biomarkers and MMPs (Lephart, 2016)	–	Bind to ERβ and activates the ERE to promote the dissociation of Nrf2 and Keap1, activate the ARE which enhance antioxidant enzymes transcription. [Female ICR mice (Jackson et al., 2011; Zhang et al., 2013; Froyen & Steinberg, 2011)]
*Polygonum cuspidatum* et al. Medical plants Grapes et al. food	Resveratrol	Increase collagen/elastin and inhibit MMPs by stimulating TIMP1, then inhibiting the decomposition of collagen. [Human skin (Giardina et al., 2010; Lephart, 2017)] Stimulating SIRT 1, extracellular matrix proteins, and antioxidants; inhibiting inflammatory and dermal‐aging biomarkers. [EFT skin cultures (Lephart et al., 2014)]	–	Upregulate mitochondrial SOD via ERβ [Human lung fibroblasts and neuroblastoma (Thornton, 2013)] Activating SIRT1 downregulation caused by UV and H_2_O_2_, protecting against UV‐ and H_2_O_2_‐induced apoptotic cell death [HaCaT/MEFs (Cao et al., 2009)] Down regulates ROS‐induced increases in AP‐1 and NF‐κB (Farris et al., 2013)
Red clover	Red clover isoflavones	Increase collagen content. [OVX rats (Circosta et al., 2006)]	–	–
*Labisia pumila*	*Labisia pumila* extract	Reduce MMP to promote collagen synthesis. [Human skin fibroblasts and keratinocytes (Chua et al., 2012)]	–	Reduce the secretion of pro‐inflammatory cytokines and protects the human skin from the ROS attacks. [Human skin fibroblasts and keratinocytes (Chua et al., 2012)]

In addition, B. Yingngam et al. studied the protection of 10 herbal extracts mediated by H_2_O_2_. The 10 herbal extracts that have estrogen‐like effects are *Pueraria candollei* var. *mirifica*, *Linum usitatissimum*, *Glycine max*, *Curcuma aeruginosa*, *Cissus quadrangularis*, *Tadehagi godefroyanum*, *Curcuma comosa*, *Butea superba*, *Trigonella foenum‐graecum*, and *Punica granatum*. The researchers found that these herbal extracts can protect cells from oxidative stress damage through their antioxidant activity. In summary, phytoestrogens protect cells from oxidative stress‐induced cell death.

## 5 DISCUSSION

Skin is a multifunctional organ, but similar to every other organ system, it is subject to both intrinsic and extrinsic aging, resulting in a loss of functional capacity. The skin provides the body with a number of functions, acting as a physical barrier between the individual and the environment, protecting internal organs from chemical and mechanical insults, and regulating metabolic activity, temperature, and water balance. It is subject to aging alongside every other organ system of the body, both naturally (intrinsic aging) and due to interactions with the environment (chronic exposure to UV, pollution, smoking, etc.) (Newton, Mcconnell, Hibbert, Graham, & Watson, 2015). Skin aging includes the loss of elasticity, a reduction in epidermal thickness and collagen content, elastic fiber degeneration, increased wrinkling, and dryness. The skin's protective function becomes compromised, and aging is associated with impaired wound healing, hair loss, pigmentary changes, and skin cancer (Thornton, 2013).

Phytoestrogens are compounds that have weak estrogenic effects in plants. Phytoestrogens exert a weak estrogen‐like effect by binding to estrogen receptors with low affinity. The molecular structures of phytoestrogens are similar to those of mammalian estrogens. Phytoestrogens are plant components with biological activity similar to that of animal estrogens and have a wide range of effects on hormone‐related diseases. Phytoestrogens are mainly derived from natural plants and foods. In recent years, phytoestrogens have become a research hotspot in the fight against skin aging (Patriarca et al., 2013; Sirotkin & Harrath, 2014; Tuntiyasawasdikul, Limpongsa, Jaipakdee, & Sripanidkulchai, 2018). Phytoestrogens can increase collagen and hyaluronic acid production and have antioxidant, anti‐inflammatory, and other effects. It is known that estrogen has a protective and beneficial effect on skin health, especially during aging and the postmenopausal period; therefore, phytoestrogens can significantly delay skin aging.

The skin undergoes degenerative changes as it ages. Skin aging can be significantly delayed by the administration of estrogen. Estrogen administration has positive effects on human skin by delaying or preventing skin aging manifestations, but the use of estrogen replacement is a risk factor for breast and uterine cancer. Phytoestrogens are natural materials with fewer side effects that have become a hotspot in research. Phytoestrogen application can improve the health of the skin and effectively resist skin aging.

The numerous mechanisms of phytoestrogens on anti‐skin aging are as follows:

Phytoestrogens can increase collagen and reduce the degradation of collagen: (1) They increase the thickness of skin collagen by inducing the expression of subcutaneous VEGF and increasing TGF‐β in the skin (Polito et al., 2012; Rona et al., 2004); (2) They reduce collagen degradation by increasing TIMP protein levels to inhibit MMPs (Giardina et al., 2010; Lephart, 2017); and (3) They inhibit FLT3 kinase activity and reduce the Ras/mitogen‐activated protein kinase/extracellular signal‐regulated kinase and Akt/p70 ribosomal S6 kinase pathways to inhibit AP‐1 activity and reduce MMP‐1 gene transcription (Park et al., 2015).

Phytoestrogens can increase the water content in skin: (1) They increase the water content in the skin by increasing EGF and HAS to increase HA in the skin (Yao et al., 2016); and (2) They stimulate an increase in glycosaminoglycans, which contribute to the water content in the skin.

Phytoestrogens can protect the skin from oxidative stress: (1) They bind to ERβ and activate ERE to promote the dissociation of Nrf2 and Keap1. Nrf2 enters the nucleus and activates ARE, which enhances the transcription of antioxidant enzymes (Kim et al., 2014); and (2) They can improve mitochondrial membrane potential and increase NO release under physiological conditions. They can also hinder the decrease in mitochondrial membrane potential and the increased NO release caused by H_2_O_2_. This protects the skin from oxidative stress (Savoia et al., 2018).

Phytoestrogens, are believed to have a wide array of benefits, whereas they, as analogues of endogenous estrogen, have been paradoxically found to promote breast cancer progression. Previous study revealed that high doses of genistein which are rich in soy products could inhibit proliferation of both ER‐positive and ER‐negative breast cancer cells, whereas low doses of genistein induced proliferation of breast cancer cells (Tang et al., 2019; Tsutsui et al., 2003). Phytoestrogens may disrupt endocrine‐dependent processes by acting as ER agonists or antagonist due to their biphenolic structure required for ligand–receptor association (Solak et al., 2014). Some researchers studied the effects of coumarin phytoestrogens on the reproductive system of mice. They found reproductive abnormalities in mice, such as early sexual maturation in females, structural and functional impairment of the reproductive system, cervical hyperplasia, inhibited expression of ERα receptors in the uterus, and reduced uterine weigh (Burroughs et al., 1990; Lee et al., 2013; Moon et al., 2009; Solak et al., 2014). Clinically, it has also been reported that phytoestrogens have some negative effects on reproduction in humans (Milligan et al., 1999; North & Golding, 2000). In addition, there are some reports that some phytoestrogens have certain side effects, such as biological estrogenic activity, and detrimental effects on thyroid function (Yue et al., 2019). In fact, the application of phytoestrogens to the skin is generally used externally. After transdermal administration, the concentration of the drug in the body is relatively low, and generally does not cause the above‐mentioned side effects. In reference to some reports expressing concern about soy supplementation and thyroid function. Sathyapalan et al., in a series of studies showed that a pharmacological dose of 66 mg of soy phytoestrogens did not increase the overt thyroid failure rate or alter thyroid function tests in patients with subclinical hypothyroidism (Sathyapalan et al., 2018). In fact, in an earlier clinical study, soy phytoestrogen supplementation in patients with subclinical hypothyroidism showed a significant reduction in insulin resistance, hsCRP, and blood pressure with little or no alterations in thyroid function (Sathyapalan et al., 2011). Finally, despite the many proposed benefits, the presence of isoflavones has led to concerns that soy may exert untoward effects in some individuals. In support of safety is the recent conclusion of the European Food Safety Authority that isoflavones do not adversely affect the breast, thyroid, or uterus of postmenopausal women (Messina, 2016).

There is increasing research on the anti‐aging effects of estrogen and phytoestrogens in the academic community. Topical administration of estrogen is also possible, but estrogen must be administered by a skilled dermatologist who can monitor the concentration and application areas to avoid any adverse effects. Phytoestrogens are natural materials with fewer side effects. Accordingly, this review demonstrates that phytoestrogens show comparable efficacy to estrogen in regard to skin aging. Phytoestrogens have an action mechanism similar to that of estrogens and exert estrogen‐like effects. However, there are still some problems that need to be further studied, such as the mechanism of phytoestrogens acting on skin, the relationship between the dosage and toxicity of phytoestrogens, the different drug delivery routes, and the absorption effect when applied to cosmetics. In general, phytoestrogens are still in the stage of development, and further research and exploration are needed in the future.

## 6 SUMMARY

In recent years, phytoestrogens have become a research hotspot in the fight against skin aging. Phytoestrogens are compounds that have weak estrogenic effects in plants. In this paper, we review the effects of phytoestrogens on human skin and the mechanisms by which phytoestrogens can alleviate the changes due to aging. This review demonstrates that phytoestrogens can increase collagen and water content and protect the skin from oxidative stress. Therefore, phytoestrogens can significantly delay skin aging.
